# Attitudes toward Female Circumcision among Men and Women in Two Districts in Somalia: Is It Time to Rethink Our Eradication Strategy in Somalia?

**DOI:** 10.1155/2013/312734

**Published:** 2013-04-18

**Authors:** Abdi A. Gele, Bente P. Bø, Johanne Sundby

**Affiliations:** ^1^Department of Social Science, Oslo University College, 0167 Oslo, Norway; ^2^Section for International Health, Department of General Practice and Community Medicine, University of Oslo, 0167 Oslo, Norway

## Abstract

Somalia has the highest global prevalence (98%) of female circumcision (FC), and, despite a long history of abandonment efforts, it is not clear as to whether or not these programmes have changed people's positive attitudes toward the practice. Against this background, this paper explores the attitudes of Somalis living in Hargeisa and Galkayo districts to the practice of FC. *Methods*. A purposive sampling of 24 Somalis, including activists and practitioners, men and women, was conducted in Somalia. Unstructured interviews were employed to explore the participants' knowledge of FC, their attitudes toward the continuation/discontinuation of the practice, and the type they want to continue or not to continue. *Result*. The findings of this qualitative study indicate that there is a strong resistance towards the abandonment of the practice in Somalia. The support for the continuation of Sunna circumcision is widespread, while there is a quite large rejection of Pharaonic circumcision. *Conclusion*. Therefore, since the “zero tolerance policy” has failed to change people's support for the continuation of the practice in Somalia, programmes that promote the pinch of the clitoral skin and verbal alteration of status, with the goal of leading to total abandonment of FC, should be considered for the Somali context.

## 1. Background

The traditional practice involving the removal or injury of female external genitals, namely, female genital mutilation/cutting (FGM/C), or female circumcision (FC), which is the term we use in this paper and is a literal translation from the Somali language (gudniinka dumarka), has long survived in Africa in the name of tradition though in recent decades the practice has received worldwide attention, with its abolishment determined by a broad international consensus [1]. Accordingly, ever more countries have incorporated the penalty against performing FC in their constitutions, and more communities and civil societies have increasingly turned their advocacy towards abolishing FC. Nevertheless, the goal remains far from being realized. With steady progress in some countries, the FC operations continue unabated in many practicing countries, thus exacerbating the already pervasive suffering for millions of women living in resource-poor countries [2]. Therefore, identifying the factors that impede the cessation of the practice may enable us to advance our understanding of the practice and the subsequent adoption of a culturally acceptable strategy towards its abandonment. 

Female circumcision is most prevalent in 28 African countries, but is also found in Asia and Western countries that host immigrants from areas with FC traditions [3]. Approximately 140 million women and girls living today are estimated to have undergone FC, with three million girls at risk Of the practice every year [4]. The motives for the practice are complex and vary between different communities, contexts, and over time [5]. These motives are all rooted in tradition and culture, though none of them carries a religious or scientific basis. The procedure is often performed on young girls by a layperson with no medical training, but in some countries health professionals performing the practice outnumber traditional practitioners [6]. A WHO study shows that 18% of all girls in countries from which data is available are cut by health professionals [7], and the operation is performed through one of four types classified by the WHO [4]: Type I involves the partial or total removal of the clitoris and/or the prepuce, while Type II involves the partial or total removal of the clitoris and labia minora. Being the most radical form, Type III involves the partial or total removal of the external genitalia and a sealing of the vaginal opening, leaving only a small hole for urine and menstrual blood to pass (whether with or without cutting the clitoris). Lastly, Type IV involves all other harmful procedures to the female genitalia for nonmedical reasons. 

The victims of this practice may suffer tissue damage that may negatively affect the health of the girls over the course of their lifetime [8]. Local and generalized infections, severe pain, acute haemorrhage, and even death may be short-term complications, with other possible complications also including the retention of urine and difficulties in menstruation [9]. A WHO study shows associations between the number of adverse obstetric outcomes and FC [8], while evidence from Norway shows that perinatal complications, such as foetal distress, emergency caesarean sections, and prelabour deaths, were more frequent among infibulated Somali women (with Type 3) compared to Norwegian women [9]. The psychological consequences following FC were described as posttraumatic shock and depression, as well as a loss of trust and a lack of bodily wellbeing [10]. 

The efforts towards the abandonment of FC date back to the early 20th century when European missionaries and colonial powers in Africa attempted to stop the practice by introducing laws and church rules, the result of which was anger against the colonial powers [11]. According to Rahman and Toubia, the governments of Egypt and Sudan passed laws on FC in the 1940s and 1950s although these laws did not work due to the absence of prior public awareness [12]. The African activism against the practice became more apparent in the 1960s and 1970s, which was the spark that motivated the WHO's first conference on FC in Khartoum in 1979. This conference and subsequent sustained activism have drawn worldwide attention to the health and human rights consequences of FC. As a result, the abandonment of all forms of FC was recommended, while the notion of reducing the physical complications of FC by performing the practice in health facilities was deemed to be unacceptable [13]. Since then, a growing number of countries have approved laws prohibiting FC [13, 14]. Nonetheless, 34 years after the first WHO conference, FC continues unabated in most high-prevalence countries in Africa, with slow progress being seen in some low-prevalence countries [2]. 

Accordingly, a number of professionals with extensive research experience on FC in Africa suggested the importance of reducing the harm when total abandonment is not feasible [15, 16]. Others stressed the importance of professional health workers performing the attenuated form of FC where the alternative is to have it performed by a traditional practitioner in an unhygienic environment [16, 17]. The harm reduction procedures include pricking, which is defined as a procedure in which the skin is pricked to draw a drop of blood while no tissue is removed [1]. Despite strong opposition by activists and international organizations such as the WHO, the data from several countries in Africa show that the milder cutting is already replacing more severe forms in many communities [18]. In those countries, the harm reduction procedure is often performed by health professionals with the intention of reducing the harm caused by the more severe procedures [19]. While the harm reduction procedure is recognized by many as a fair intermediate step offering safer solutions in the process of change in areas where total abandonment is not feasible, a joint statement by the WHO and other UN agencies raised concerns that pricking (if it is accepted) may serve as cover-up for more invasive procedures [1]. 

The country with the highest prevalence of FC in the world is Somalia, where virtually all girls in the country are circumcised (98%) [20]. The practice is often performed on Somali girls between the ages of 4–10 by a medical practitioner, or most often by a traditional practitioner from a family in which generations of that family have been traditional practitioners. Somali people classify female circumcision into two types: the Sunna form (*gudniinka sunniga ah),* which is perceived as mild, but can encompass anything less than Pharaonic, and the *Pharaonic* form (*gudniinka fircooniga ah), *which is considered to be severe and involves suturing the side fleshes together, leaving a small opening for urine and menstrual blood to pass through [21–23]. The term “*Sunna,*” literally means “tradition” in Arabic. For Somalis though, the word “*Sunna*” means any tradition of the Prophet Mohamed that his followers preserve. As a result, many Somalis may opt for this form, subsequently becoming resistant to abandoning any practice that carries the name “*Sunna*” [24]. 

 The circumcision of both girls and boys is equally perceived as a normal aspect of being a Somali [24], and the age range of circumcision is similar among both Somali boys and girls (<10 years). It is the mother's duty to make arrangements for the circumcision of daughters, whereas the father is expected to organize the circumcision of their sons. The rationale behind this is that young girls may not be eligible for marriage if left uncircumcised. Like many other societies in which women have limited access to education and employment [25], marriage in Somalia is critical for women's economic security. Thus, ensuring that a daughter undergoes circumcision is a loving act aimed not only to boost a girl's chance of a successful marriage, but also to promote integration into her culture. A failure to circumcise daughters may result in a long-lasting stigma and shame on the girl and her mother. The word “*buuryo qab*” (uncircumcised), which is the worst kind of insult a Somalian can hurl at another Somali, is frequently said to the daughter by her age-mates, with this applying to boys as well. Regardless of gender, being uncircumcised therefore remains outside of the accepted Somali cultural standards. 

Beginning in the 1970s, the former Somali regime openly took a stance against FC and backed numerous campaigns aimed at eliminating the practice using a variety of approaches. The Somali Women's Democratic Association (SWDA) was founded in 1977 to implement anti-FC projects with overall goal to abolish the practice in Somalia by the year 2000 [26]. One of the recommendations by SWDA was a promotion of the pricking form to be performed in hospitals with an expectation that this milder form will eventually replace infibulations [27]. Unfortunately, since people had no prior awareness, this strategy did not work. Lastly, the practice was banned from hospitals, and the total abandonment strategy was adopted in 1988 [27]. The Somali government and Swedish agency (SAREC) provided support to the Somali Academy of Science and Arts to engage in research on the topic. Simultaneously, knowledge creation campaigns were initiated in schools and community meeting centres, and a nationwide “media-based” campaign was launched. All government efforts against FC collapsed with the overthrow of the military regime in 1991, and decades of conflict in the country interrupted not only academic research on the topic, but also halted any effort to coordinate a national action plan of any kind [28]. Since 1996, though, some uncoordinated programmes on FC have been implemented in different parts of the country by both international and local women organizations. Most of these efforts have been based on awareness-raising lectures and seminars at the community level, in which the health and human rights risks associated with the Pharaonic cut have been discussed. However, the use of high-level advocacy activities to create a social and political atmosphere that rejects all forms of the practice has been very scarce. Some years ago Tostan, a civil society organization that made a significant improvement in FC abandonment in Senegal [29], also replicated its FC abandonment programme in villages in Somaliland, the same region where the present study was conducted. However, according to a film entitled, *“My daughter, dry your tears,”* which was made as a result of a Tostan programme in Somalia, the programme seemed to have maintained the status quo by rejecting the Pharaonic cut only. A prior assessment report on FC programmes in Somalia shows that programmes “lack systematic approaches, strategies, consistent messages and appropriate materials” [23]. Thus, the question of whether or not these programmes have changed the predominantly positive attitudes of Somalis toward the practice of FC remains unanswered [23]. 

## 2. Behavioural Change Theories

When people lack an awareness of how their behaviour affects their health and wellbeing, they have little reason to put themselves through the misery of changing the risk behaviours they have engaged in for many years. Although increased knowledge creates a precondition for change, yet additional communal or self-influences are needed to overcome the impediments to adopting and maintaining new behaviours. There are large numbers of behavioural change theories, but changing the behaviour of FC requires a unique approach, as it is a communal rather than individual behaviour. One of the main characteristics of FC is that even if each individual in the intermarried group thinks of abandoning the practice, no single individual acting alone can succeed [30]. According to Mackie, the best possible way to achieve a successful change is to accomplish a convention shift of intermarrying communities and a public declaration that marks the shift, in which every family understands that FC is harmful. Nevertheless, there is no single family that can act alone, as a group of intermarrying families is required to abandon the practice simultaneously [30]. Convention theory illustrates that where all families in an intermarrying society choose not to have their daughters circumcised, cutting would not be an incentive to any family's daughter [30]. This situation has been seen among Somalis in Norway [31, 32]. However, in the absence of a collective shift, the practice will continue even if every person wants to stop it on an individual basis [30]. The most successful programmes that have led to a reduction in the prevalence of FC include the Tostan programme in Senegal [29]. Moreover, comprehensive approaches adopted by Norway for the abandonment of FC led to a significant attitude change among Somali immigrants in Norway [31, 33], with other European countries also reporting similar results [3, 34]. In Somalia, there are a number of FC programmes underway, including the Tostan project in Somaliland. Even so, there is no evidence as to whether or not these programmes have changed people's positive attitudes toward the practice of FC. This study may be useful in helping to better understand the current attitudes of the Somali people toward this persistent but widely criticized practice, in addition to creating discussions of possible, context-specific alternatives to help minimize the suffering due to FC, as well as to ultimately abolish the practice. 

## 3. Materials and Methods 

### 3.1. The Context

Somalia is a Horn of African country with population of 7-8 million, of which approximately 20–30% live outside their home country, with majority of them living in Western countries. Roughly 44.7% of Somalis are between the ages of 0–14 years, the total fertility rate is 6.26 children per woman, and the literacy rate is 49.7% for males and 25.8% for females. While infibulations, which are associated with various obstetric complications [9], constitute 85–90% of all types in Somalia, women who give birth in hospitals and receive professional care during the delivery constitute only 9% of all total deliveries in Somalia [23]. Accordingly, a prior study shows an extremely high maternal mortality rate of 31 deaths in 734 deliveries in Galkaayo, Somalia (4,223 in 100,000) [28], with all the women in this study being victims of infibulations. The infant mortality rate in Somalia is 180 per 1,000 live births, while the maternal mortality ratio is 120 per 100,000 live births [35]. 

### 3.2. Study Design

A qualitative study using unstructured interviews was conducted in Galkaayo and Hargeisa from July to October of 2011 and May of 2012. An unstructured interview is not only a flexible tool for accessing people's experiences, inner perceptions, attitudes, and feelings of reality, but also a tool for understanding the complex behaviour of people without imposing any a priori categorization that might limit the field of inquiry [36]. Gaining trust and establishing good relationships are essential to the success of unstructured interviews, as only when a trustful and harmonious relationship is cultivated the interviewee can share his/her knowledge and experience on sensitive topics such as FC with the interviewer.

A purposive sampling of 24 Somalis aged ≥18 was conducted in Somalia. Of the participants, 11 were men and 13 were women. Among them were two activists against FC from local NGOs and two trained midwives, who at the same time served the community as circumcisers. The remaining 20 participants were ordinary people from various walks of life, but all of them were part of an urban population.

We followed common research ethics principles in the carrying out of this study, including informed consent, the right to refuse, withdrawal, and confidentiality. Afterwards, verbal consent was obtained from each participant, and this study was ethically cleared by both the Norwegian Ethical Committee and the Ethical Committee of the Ministry of Health of Somaliland. 

The topic of FC is not a sensitive topic, nor is it illegal in Somalia as it is in Western countries; hence, people openly expressed their experiences and perceptions towards the practice. However, the discussion of FC between males and females is rare. Thus, to reduce gender sensitivity related to FC, we assigned a female assistant to interview the female participants and a male assistant to interview the men. Interviewers received training about how to conduct qualitative interviews, as well as how to develop trust and intimate relationships with participants prior to the initiation of the interview. Moreover, they were given information about the study's objectives, in addition to the research question that the study intended to answer. Activists and midwives were interviewed by the first author, and two days were set aside for each participant, the first day for getting to know one another and building a relationship and the second day to inform them about the study and to subsequently obtain their consent for participation. Being a male interviewer did not affect the quality of the interviews of female midwives and activists since the participants had to talk about their professional experience on the subject, but not their personal situations. The interviews were conducted in the Somali language, which was the native language of the participants and interviewers. 

### 3.3. Content of the Interview

During the interviews, the terms *gudniinka dumarka *(female circumcision), gudniinka sunniga (milder forms), and *gudniinka fircooniga* (infibulation) were used, and the participants were asked about their understanding of the practice, as well as their perspectives regarding the continuation/discontinuation of the practice. They were also asked if they circumcised their daughters or had intentions to do so. The participants' denotation about the Sunna and Pharaonic cut was also explored, and the interview process continued until it was clear that no new information was emerging from the additional interviews; that is when saturation was achieved.

### 3.4. Analysis

The first author translated the audiotaped interviews into English and transcribed them verbatim. The transcripts were thoroughly read several times [37]. We used a thematic analysis to identify and analyse important themes [38], with the coding process involving the recognition and encoding of the identified themes prior to interpretation [39]. According to Leininger [40], themes can be identified by bringing together fragments of ideas, experiences, and beliefs that are often meaningless when viewed alone. For that reason, themes that emerged from the informants' stories were pieced together to form a comprehensive picture of the participants' shared experience [41]. The themes that were identified through coding were further divided into categories based on the participants' experience, knowledge, and attitude towards FC [42]. The consistency of the findings from different methods we used (i.e., interviews and quantitative data that were published elsewhere) has served to ensure the trustworthiness and credibility of the study's results.

## 4. Results

### 4.1. Participants' Knowledge on Types of FC and Its Health-Related Problems

The majority of the study participants divided the types of FC as Pharaonic (Type 3) and *Sunna*. *Pharaonic* was perceived as the procedure involved in cutting most of the external genital tissue, with the sides fused together to leave only a small opening. By contrast, the *Sunna* was perceived as the milder form that does not lead to any health-related problems. However, some participants mentioned Sunna circumcision as having two stitches, which generated a motivation to interview the circumcisers to help explore the extent of the cut entailed in the Sunna circumcision. They described two types of FC that they perform:

*There are two types of Sunna. One is where a drop of blood is obtained from the clitoris, or the tip of the clitoris is incised, while the other type is called Kaatun (ring), which most commonly involves the removal of the prepuce. The clitoris is either removed totally or partially, and then two stitches are made. (A 59-year-old female circumciser)*

The circumcisers' information is supported by activists who reported that what people perceive as Sunna can be categorized as Type 3 because of the involvement of the cutting of different tissues:

*There are no people who are trained for doing Sunna circumcision. The same people who used to do the Pharaonic are also doing the Sunna. They cut all the parts that they used to cut in the Pharaonic. The only difference is whether they suture less or not. (56-year-old female activist)*

### 4.2. Knowledge on the Health and Human Rights Consequences of the Practice

The vast majority of the study participants knew that FC had health-related problems, including recurrent pain, the retention of urine and menstruation, and infection and complications in childbirth. Moreover, fistula, as well as sexual dissatisfaction and psychological problems, was also mentioned, whereas some participants attributed the frequent school absenteeism of the girls to FC. Even so, none of the participants blamed the Sunna form for any health problems, while most of the participants attributed all health complications due to FC to Pharaonic circumcision:

*Pharaoanic circumcision creates lots of problems for girls; … they are sutured during the operation, they are defibulated at their first marriage, they are defibulated again when giving birth. What kinds of benefit can it have? Nothing! It has only disadvantages. (50-year-old female) *

*There are lots of problems, that is, in the Pharaonic form, girls have difficulties in passing urine and menstrual blood. Urine may keep draining for a long period, because urine that is retained under the sutured flesh may keep draining long after the initial urination. (35-year-old female)*

*It creates so many problems, including blood retention, urine retention, difficulties in child birth and other problems. (26-year-old male)*

*When the passage for urine, blood and childbirth is closed, complications are unavoidable. Sometimes fistula develops, which is very serious. Moreover, men experience problems when they marry a woman who was circumcised with Pharaonic. (41-year-old male) *

Some participants acknowledged that FC has adverse health effects on the sexual satisfaction of women, which they perceived to be a problem:

*Uncircumcised girls have their natural feelings (sexual satisfaction), but the circumcised ones have lost their natural feelings. (20-year-old female) *

*Circumcision destroys the sexual life of girls, and I believe it affects them psychologically too. (25-year-old female)*

The potential impact of FC on girls' education was also recognized by some of the participants, while the high level of school dropouts and school absenteeism among Somali girls was also attributed to the practice:

* If the girl goes to school, she may miss class at least one week every month because of problems associated with Pharaonic circumcision. (43-year-old female)*

*Pharaonic circumcision causes pain that sometimes stop girls from going to school. (36-year-old male) *

Moreover, the participants knew that FC was a violation of human rights of women and girls. They mentioned that cutting natural female genitals is unreligious and ruins the health of the women, therefore violating the rights of girls to a healthy life:

*You have a responsibility. You should treat girls according to the Islamic religion, you do not have to harm them. (32-year-old female) *

*I think every person whether young or old has a right for his/her natural body to not be mutilated. If there was an advantage to mutilating the body of women, God wouldn't have created those tissues in the first place. (28-year-old male)*

*Every person has a right to health; when his/her right to health is ruined by cutting important parts of the body, that is a violation of human rights. (37-year-old male) *

### 4.3. Circumcision Status of the Participants' Daughters

Despite a good amount of knowledge on the health and human rights consequences of FC, none of the participants had any intention to leave his/her daughter uncut. The majority reported having subjected their daughters to the Sunna form, which they perceived as being similar to being untouched:

*Now I did not suture my girls, I only circumcised them with the Sunna form, and I thank God that they did not experience all the pain associated with the Pharaonic form. (44-year-old male)*

*I have two daughters and I subjected them to a minor Sunna and I did not touch them. (35-year-old female)*

*I have two daughters. They are still too young to be circumcised, but I will circumcise them with Sunna circumcision. (28-year-old female)*

*I have girls and I circumcised them with the Sunna form. (39-year-old female)*

Nonetheless, three participants reported having their daughters circumcised with the Pharaonic form, though one mother mentioned having her daughters circumcised with Sunna and subsequently with the Pharaonic circumcision:

*I have five daughters. At the beginning, me and my husband disagreed about the type of circumcision for the girls, as he wanted the Sunna form. I accepted his suggestion and the first two daughters were circumcised with Sunna, but after some months with the Sunna circumcision, I took them back to the circumcisers and they were sutured. I circumcised the other three girls with Pharaonic circumcision too. (41-year-old female) *

* I have four daughters and I will circumcise them with the Pharaonic form. (33-year-old female)*

*I have a daughter and I circumcised her using Pharaonic with three stitches. More than three stitches is impossible. (20-year-old female)*

### 4.4. Resistance to Total Abandonment

Almost all the participants in this study supported the continuation of FC in one form or another, admitting that the tradition of leaving girls untouched has no room in their culture. However, they supported the *Sunna* cut, which they believed to be a religious requirement with no health-related problems:

*Untouched girls!! That is too much and impossible here. The message should be very clear; girls must be circumcised with Sunna, which is a religious duty. But, girls will not be circumcised at all! That is a very strange story here. (38-year-old female)*

*In our culture there are no uncircumcised girls. Girls should be either circumcised with Pharaonic circumcision or the way the religion accepts (Sunna). (40-year-old male)*

*We are people who have a long history of circumcising girls, we moved from the Pharaonic to a milder form and further to the mildest form. I think if total abandonment is suggested, there is nobody who is going to accept it. (35-year-old female)*

*I do not support the total abandonment of FC, but I want the Pharaonic type to be abandoned. Girls should get the mild Sunna. It is harmless and it does not interrupt the daily work of girls. (35-year-old female)*

### 4.5. Justifications for the Continuation of FC

Different justifications were put forward for the defense of girls' circumcision. Those who were sympathetic to Sunna circumcision forwarded totally different arguments for the continuation of the practice than those who were supportive of the Pharaonic form. Religion was the main justification for the continuation of the Sunna form, while the belief that Sunna circumcision has no health-related problems was also widespread:

*Our old generations used to circumcise our daughters with Pharaonic, but recently people have abandoned Pharaonic because they came to know that religion does not accept the Pharaonic. I support the Sunna form because it is good for religion and it does not cause harm. (28-year-old female)*

*In the old days (with Pharaonic circumcision), girls were used to being on the bed for eight to nine days with their legs tied together. But the Sunna form has no problems at all. Girls are cut and they go without any problems. (39-year-old female)*

Many of the participants rejected the Pharaonic circumcision, as it is perceived as being un-Islamic and harmful to one's health. Yet some women believe that the Pharaonic cut has to be continued regardless of its un-Islamic nature and its adverse effect on the health of girls and women. The main reason they forwarded was its potential for virginity and marriageability:

*Pharaonic is a crime according to our religion and it is not allowed, but every person has his/her choice, I always support Pharaonic and I still support it. Let God punish me for that if God wishes. In my neighbours and relatives, I have not seen a single girl that is left with only Sunna circumcision (all are cut in Pharaonic). (20-year-old female)*

*Islam does not accept Pharaonic circumcision. But each individual does what he/she thinks is safer and good for his/her daughters, and we feel that Pharaonic is more secure for us. I know what is good for my daughter, it is my responsibility to do it. (41-year-old female)*

*I believe the former type (Pharaonic) was better, the Sunna form is not good. In the Sunna form, there is no difference between old women and girls regarding virginity because both are open. When a mother of 10 children and a young girl cannot be differentiated regarding virginity, as both are open, it is a big shame. I support the Pharaonic form and I encourage mothers to subject daughters to Pharaonic. (20-year-old female)*

### 4.6. Obstacles to Total Abandonment of the Practice 

#### 4.6.1. Suspicions Surrounding the Abandonment Programmes

Activists told about the factors they believe are obstacles toward a successful attitude change towards FC, with their stories revolving around two issues. First, people are suspicious about the social change itself, which they think is a foreign-driven agenda that is offensive to their religion and culture. Secondly, the practice of FC remains as a strong social convention that traps everyone in the society, and no one can escape the trap alone; if so, that person will pay a steep price:

*People are defensive; they say that these people (NGOs) want to change our religion. Others say that if we stop FC they will again tell us to stop men's circumcision, and then they will tell us something more unusual. (47-year-old female activist)*

*In 1991, we performed the first awareness campaigns, and I reflect on a young girl whose mother decided not to circumcise her. After 20 years when the girl got married, she was divorced immediately after her husband realized that she was not sutured. She was circumcised later at the age of 20. So, there are suspicions. The question people ask is: How can I trust that my daughter will have a successful marriage if I do not circumcise her? (56-year-old female activist)*

#### 4.6.2. Uncooperative Opinion Leaders

One of the main obstacles toward the total abandonment of FC that emerged from the interviews was the fact that opinion shapers, such as religious leaders are sympathetic to Sunna circumcision, and therefore encourage its continuation:

*After people understood the health problems of FC, they asked religious leaders about the position of Islam on FC, and they were told to use the Sunna circumcision. We invited some Somali religious leaders to Saudi Arabia, where they were told that FC has nothing to do with Islam. When we came back home, they insisted that the Sunna should not be stopped. People said that if we abandoned Pharaonic circumcision, then let us keep doing the Sunna. (56-year-old female activist)*

*We invited highly academic religious leaders from Alazar[sic] University in Egypt to come here, and they supported the total abandonment of FC. Somali religious leaders did not accept that by saying that the Sunna form should continue. (47-year-old female activist)*

#### 4.6.3. Awareness Programmes That Reject Only One Form

In response to a question about “whether the participants have attended awareness programmes and what they have learned from the programme,” almost all the participants who attended awareness programmes or heard about it on TV were told to abandon Pharaonic circumcision. Some participants stated that they were told to circumcise girls with Sunna circumcision and abandon Pharaonic:

*Yes I attended seminars. I was informed that the Pharaonic form should be abandoned because it is wrong. Girls should be subjected to the Sunna form, which is not harmful to girls. (35-year-old female)*

* I attended seminars…I have learned that Somalis should abandon the Pharaonic circumcision, as it causes a number of health problems. (21-year-old female)*

*I attended seminars and I learned about the problems of Pharaonic circumcision. (26-year-old male)*

*I did not attend a seminar, but I heard from the radio and the media that Pharoanic[sic] circumcision should be stopped. (31-year-old male)*

#### 4.6.4. Absence of a Law against FC

Participants were asked about their perspectives toward the criminalization of FC in their country. The vast majority of the study participants stated that the criminalization of the practice would not be a solution, but that it may instead create a confrontation between the government and the public. The illegalization of Sunna circumcision is perceived as a violation of religious rights and a serious issue that cannot be tolerated:

*If the government accepts that girls are circumcised with Sunna circumcision, people will accept it, but surely people will not accept total abandonment. Rejecting the Sunna form that religion requires is like saying “abandon your religion”. (39-year-old male)*

*This is a strong tradition that existed for a long time. If it is intervened through the legal system, it will grow even stronger. People may say that the government is against our culture, tradition and religion. This may put the government at risk. (22-year-old male)*

*I would not support illegalization because if the government gets involved with this long tradition, while very few people shifted to Sunna and the majority still used Pharaonic, the government will have difficulties. I think they should wait until more people shift to Sunna. People should be encouraged to abandon the practice and be given knowledge about it. But the government and NGOs should leave the decision about its abandonment to the people. (30-year-old male)*

## 5. Discussions and Conclusions

This qualitative study explored the attitudes toward FC among Somali men and women in the Hargeisa and Galka'ayo districts of Somalia. The findings show that almost all the participants supported the continuation of female circumcision, with the majority supporting the continuation of the* Sunna* form in particular, while rejecting the *Pharaonic *form. This finding is consistent with prior quantitative findings in Somalia [43, 44], in which 90% or more supported the continuation of the practice, particularly *Sunna* circumcision. Given the fact that many Somalis may support one form of FC while rejecting others, the importance of the categorization of questions addressing FC by type is underlined; a failure to do that may cause a risk that researchers will draw the wrong conclusions. For instance, the Multiple Indicator Cluster Survey (MICS) by UNICEF in 2006 demonstrated that only 32% of people in the northwest region of Somalia support the continuation of FC [20], which contradicts virtually all of the other studies conducted in Somalia. The difference between the MICS and other studies could be the way the questions were formulated, as the MICS asked the people about their support in relation to the continuation or discontinuation of FC, while our study categorized the practice into *Sunna* and *Pharaonic, *asking them which one should be continued or discontinued. The MICS also considered the categorization of the practice by type in their published questionnaire although they did not consider the answers to this question in their report [20]. 

The present study explored the extent of the cut involving the *Sunna* circumcision in Somalia, with the findings revealing that the most prevalent Sunna cut in Somalia involves either the partial or total removal of the clitoris, followed by two stitches. When the operation involves stitching, the WHO classifies it as Type III regardless of the number of stitches [4], which is consistent with prior findings that reported that most Somali parents ask their infants to undergo the larger excisions [28]. Some of the participants who defended the continuation of the Sunna cut defined the “Sunna cut” they advocated for as the “pricking of the clitoris.” Nevertheless, none of the participants clearly indicated to have his/her daughter subjected to this mild type. Prior studies on FC among both Somalis in exile and other similar communities argued that circumcisers may claim that they performed Sunna when they really performed a more extensive form [32, 45]. At this stage, we do not know whether or not the circumcisers decide what to cut and what not to cut, or whether it is the parents who should regulate the extent of the cut to be performed by the practitioner. An interview with circumcisers reported that the mild form involving the “prick of the clitoris” exists in their setting, but is rare. Through my personal experience, if you ask a Somali about the definition of the Sunna cut that religion accepts, virtually everyone may say “the pricking of the clitoris.” However, the findings of this study make it clear that the Sunna form that people in Somalia might have shifted to is not as mild as we may once had thought. 

This study reports a pervasive resistance to the total abandonment of FC in Somalia. The majority of the participants in this study supported the continuation of Sunna circumcision, while few people supported the continuation of Pharaonic circumcision. The Sunna cut may involve anything different from the Pharaonic cut, and behind the support of the Sunna circumcision lies the belief that Sunna circumcision is a religious requirement. Generally speaking, supporters of Sunna circumcision use a single Hadith as a justification for their argument. The hadith says, “*Do not cut too severely, as that is better for a woman and more desirable for a husband.*” However, many religious scholars regarded this passage as having little credibility or authenticity. Even so, the Koran clearly rejects an alteration of the human body from the way God has created it. Female circumcision is therefore a controversial topic within Muslim circles; still, the important point to note is that Islam safeguards women's rights to sexual enjoyment and health, and if female circumcision violated those rights it would automatically be considered as being forbidden. 

The participants who supported the continuation of Pharaonic circumcision used its importance for the virginity and marriageability of girls as a justification for the continuation of FC though it is unfortunate that the practice designed to make girls reproductive (marriageable) may ultimately cause them to become infertile [46]. While being fully aware of the health consequences of the practice, as mothers themselves have gone through the adverse consequences of the practice, they still subject the same procedure on their daughters. In a country where almost all the women have been circumcised, being uncut has become a social stigma, as uncut woman may have little chance of getting a husband. Thus, it is not surprising that there is pressure by mothers and relatives to allow their daughters to undergo female circumcision [47]. The theory of reasoned action and the theory of planned behaviour argue that before individuals change their behaviour, they often consider the consequences of the change [48]. The health consequences of FC are vast, but it is disproportional to the adverse social and cultural consequences that, if left uncircumcised, girls and their families may endure. This is the reason that despite a very good knowledge of the health consequences of FC, all of the participants who had daughters reported that they either subjected their daughters to FC or had the intention to do so. The question then that programme leaders must ask themselves before designing any intervention is how much control does a mother have in stopping her daughter from circumcision? In the case of FC in Somalia, no single individual has control over their behaviour concerning FC since the practice is a deeply believed social norm. In such a social environment, knowledge creation and awareness campaigns alone, as the situation has been for the past 30 years, may not be enough to change the status quo. 

This study shows the number of obstacles toward the abandonment of the practice in a study setting, with the most important thing being the support and tolerance of the practice by opinion leaders. The importance of religious leaders/politicians' involvement in the abandonment of the practice has been documented [14]. However, the information received by Somalis from religious institutions regarding FC is that the “*Sunna* circumcision is allowed by religion and it should be continued” [49], as the political leaders are either silent or support the continuation of the practice. The recent objection against the illegalization of all forms of the practice by parliamentarians in the Puntland state of Somalia was a clear indication of the widespread tolerance of the practice at the political level. In addition to the rejection of illegalizing the practice, those who brought the case in the Parliament were alleged to have violated the basic religious values of the community until they eventually pled that “they meant to abolish Pharaonic circumcision only.”

This study also indicates that campaigners in Somalia reject the Pharaonic form only, and not the Sunna form. Hence, it is important to note that FC is a strongly believed in tradition, and that whoever dares to oppose it in Somalia does so against the tide of public sentiment. The campaigners themselves are part of society and cannot go against the will of the public or they may pay a heavy price. It is therefore very clear that the public rejection of the Pharaonic cut is widely prevalent in Somalia while the Sunna cut, which is anatomically undefined, entertains overwhelming support because of its association with religion and the perception that the Sunna cut does not have any health problems. Shell-Duncan states that “harm reduction strategies may be a sound and compassionate approach to improving women's health in settings where total abandonment of the practice is not immediately attainable” [16]. The author used the example of other widely accepted harm reduction strategies such as the treatment of heroin addicts with the use of methadone, which improves the health of heroin addicted individuals, and since it is orally administered, it also reduces the risk of blood transmitted diseases. Shell-Duncan concludes that “a harm reduction approach shares the goal of eventually eliminating female genital cutting, but is willing to promote intermediate steps that offer safer solutions in the process of change” in areas where the more severe form of FC is predominant and total abandonment is not feasible [16]. According to Yoder et al., FC practicing families live in three different social environments: (1) “those in which nearly everyone has their girls cut, (2) those in which no one has their girls cut, and (3) those in which some girls get cut while others do not” [18]. Families who live in the latter two social environments are more likely to give up the practice through zero tolerance-based interventions, as they interact with communities who do not circumcise their daughters, thus possibly inspiring them to abandon the practice. When Somalis migrated to countries such as the UK, Sweden, and Norway, many of them give up the practice because FC is not embraced by the mainstream communities in the host countries or with other Muslim immigrants such as those from Pakistan [3, 33, 50]. 

In 2011, the WHO published a policy brief entitled, Female Genital Mutilation programmes to date: what works and what does not [51]. The report concluded that the “interventions that worked included those which involved coordination between NGOs and governments” in which governments adopted laws against the practice. The report neglected to answer the question: what works for whom? The strategy that can work in communities located in conflict areas where there are no effective governments, such as Somalia, was not considered in the report. Nonetheless, the generalization of highly diverse contexts, communities, and cultures has been the reality over the last three to four decades. In reality, the strategy that works among Somalis in Norway may not work in the Somali community in Somalia because the two contexts are different. Similarly, the strategies that work in the Kisii community in Kenya may not work in the Somali community in Kenya, since almost everything surrounding the practice is different in the two cultures. Therefore, the total abolishment of FC in Somalia, where there is no effective government, requires a context-specific strategy designed solely for the Somali community in Somalia. 

Recently, the Public Policy Advisory Network on Female Genital Surgeries in Africa published a policy report entitled, “Seven things to know about female genital surgeries in Africa” [52]. This policy brief has opened the door for a broader debate about the current global policy on FC, thereby highlighting the failure of FC abandonment by conservative policies, such as a “zero tolerance of FC,” which have been based on the notion that “this way (zero tolerance) or no way at all.” According to the report, the “zero tolerance of FC” slogans closed the door on the diverse discussions that have been a precondition in finding solutions for this age-old practice that affects the health of millions of women all over the world [52]. According to Shweder, there are discrepancies between the global discourse and the experience of many field researchers in Africa [53]. In an effort to minimize health risks due to more extensive forms of FC, several proposals for harm reduction methods have been developed. Mathews stated that the Royal Australian and New Zealand College of Obstetricians and Gynecologists (RANZCOG) considered the sanctioning of the medically performed pricking form to prevent the more severe procedures, but after harsh criticism from activists the decision was revoked [54]. Similarly, the American Academy of Pediatrics' Committee on Bioethics endorsed the importance of ritual nick to save some girls from undergoing disfiguring and life-threatening procedures [55]. Later on, the decision drew storms of criticism from activists and was subsequently revoked [56]. Whenever there has been a proposition of an alternative procedure by researchers or other professional institutions such as pediatrics and gynecologists, a fierce criticism by activists arises. We all respect the human rights of women and children, and we endeavour to find ways to put this practice to an end. However, there is a discrepancy in the understanding of the process leading us to the abolishment of this age-old practice. It has been over three decades since we began advocating for a zero tolerance of FC, with the progress made being far from the desired extent. Yet, despite several recommendations for the pricking form as a transition to total abandonment, no chance has been given to testing the impact of such an intermediate step for the abandonment of FC in countries where zero tolerance has failed. 

This study shows that both ordinary people and opinion leaders in Somalia are against the total abandonment of FC but supporting for the continuation of the Sunna form. Campaigners against the practice could not dare to also promote zero tolerance slogans since this goes against the will of the public majority. The Somali government adopted a zero tolerance strategy in 1988 after the pricking form strategy failed though it did not work well [27]. As mentioned earlier, high-profile government ministers who attempted to promote zero tolerance in the Puntland state of Somalia in early 2012 faced serious opposition from Parliament, and a threat to lose their government positions, until they finally revised their claim. This clearly shows that the zero tolerance strategy has been attempted several times in Somalia, but failed through strong public resistance. Accordingly, our recent quantitative study of the urban population of Hargeisa (soon to be published) shows a prevalence for FC of 97%, with over 80% of the women being infibulated. This is consistent with studies that were conducted in early 1990s [57], and it is an indication that girls' circumcision, particularly with Type III, continues unabated. Hence, after 30 years of a failed strategy in Somalia, shall we still wait and watch the pain and suffering endured by thousands of girls who are infibulated every year until the zero-tolerance strategy works in Somalia, or should we open the doors for discussions and alternatives? The time is right for local communities and donors to act decisively to support what is working to end female circumcision in Somalia.

For many ethnic groups in Africa, FC represents the central component of a traditional rite of passage ceremony in which girls are expected to pass through a transition from puberty to adulthood. In these communities, in making the decision to not circumcise their daughters, parents may face the dilemma of what to do about the traditional ritual that allows them and their daughters to publicly announce the transition to womanhood. To address this problem, the idea of an “alternative ritual,” which excluded genital cutting but maintained the ceremony and the public declaration for community recognition, has been adopted. A symbolic ceremony strategy without genital cutting has been reported to become successful in parts of Kenya [58]. Thus, a similar strategy tailored to Somalis' understanding about the practice is crucial for the future abolishment of female circumcision in Somalia.

This study has some limitations. The results of the study reflect the perceptions of a limited number of participants in the study, and not necessarily those of the entire Somali population in Somalia. The failure to generalize the findings of this study to the Somali community in Somalia is a general limitation of the qualitative methods used. Most of the views and opinions were repeatedly expressed among different individuals, and the result is consistent with our earlier quantitative results in Hargeisa, thereby increasing our confidence in the validity of the findings.

In conclusion, will the promotion of the pinch of the prepuce and verbal alteration of status may be a better strategy for the eventual abandonment of FC in Somalia? The idea is not to promote a shift to a milder form of FC, but to formulate a harmless situation that mimics a circumcision through a transition to untouched behaviour. The pinch of the prepuce will alleviate the suffering due to Type III, Type II, and even Type I, while it may be much less painful than widely tolerated practices such as genital piercing, ear piercing, and male circumcisions [59]. The advocacy for the pricking form may not be an option in areas where total abandonment can be achieved through zero tolerance. However, it may be a fairly temporary solution (as a transition to total abandonment) in areas where there is no immediate feasibility of total abandonment, and infibulation is the most common procedure [16]. 

There are a number of assumptions for the potential of this strategy in abolishing FC in Somalia. First, the “the pinch of the clitoral hood” may break the religious argument surrounding the practice, which is the most important argument for the continuation of FC among Somalis, and it may neutralize the religious leaders' opposition to total abandonment. Nonetheless, it is important for programme leaders to avoid using the term “Sunna” when promoting this mild form, as detaching the practice from religion is crucial in any effort towards the total abandonment of FC in Somalia. 

Secondly, it will break the link between marriageability and FC, which is the second main reason for perpetuating FC in Somalia. In this case, the association between virginity (marriageability) and FC will eventually disappear as the Pharaonic, which has been perceived as guaranteeing virginity, will be eliminated through public consensus. Down the road, there is an expectation that people will gradually adopt uncut behaviour, as the pinch of the clitoral hood will no longer be seen as being necessary for virginity and marriageability; nor will people (peers or the groom) have a way to differentiate a girl with a pinched clitoral hood from an untouched girl. Thus, those who were already against the practice, but had it performed on their daughters because of the social pressure, may automatically abandon the practice at all.

Thirdly, it may give a reasonable voice to activists to advance their advocacy against the practice, since they can easily convince politicians and religious leaders to be on board in their advocacy towards the abandonment of all other forms of FC. It is an idea that every person in Somalia can easily digest since the majority of Somalis are convinced that Pharaonic circumcision and all forms involving the cutting of tissues are neither religious nor a good part of the culture. The bottleneck for the past 30 years has been the notion that girls should be left untouched, which can be neutralized by adopting this circumcision mimicking form. The prerequisite here is that programme leaders should have a clear mind in terms of what is to be promoted (the pricking of the clitoral hood), such that the pricking form should not serve as a cover-up for more severe forms of the practice. 
